# Formation of Nb_2_O_5_ matrix and Vis-NIR absorption in Nb-Ge-O thin film

**DOI:** 10.1186/1556-276X-7-341

**Published:** 2012-06-25

**Authors:** Seishi Abe

**Affiliations:** 1 Research Institute for Electromagnetic Materials, Sendai, 982-0807, Japan

## Abstract

This paper investigates the crystal structure and optical absorption of Ge-doped Nb-oxide (Nb-Ge-O) thin films prepared by RF sputtering. A wide-gap material, Nb_2_O_5_, is selectively produced as a matrix to disperse Ge nanocrystals through compositional optimization with Ge chip numbers and oxygen ratio in argon. The optical-absorption spectra are obviously shifted to visible (vis) and near-infrared (NIR) regions, suggesting that a composite thin film with Ge nanocrystals dispersed in Nb_2_O_5_ matrix exhibits quantum-size effects. Accordingly, the two valuable characteristics of the Nb_2_O_5_ matrix and the vis-NIR absorption are found to be retained simultaneously in Nb-Ge-O thin films.

## Background

Quantum-dot solar cells have attracted much attention because of their potential to increase conversion efficiency [1]. Specifically, the optical-absorption edge of a semiconductor nanocrystal is often shifted due to the quantum-size effect. The optical band gap can then be tuned to the effective energy region for absorbing the maximum intensity of the solar radiation spectrum. Furthermore, quantum dots produce multiple electron–hole pairs per photon through impact ionization, whereas bulk semiconductor produces one electron–hole pair per photon.

A wide-gap semiconductor sensitized by semiconductor nanocrystals is a candidate material for such use. Wide-gap materials such as TiO_2_ and ZnO can only absorb the ultraviolet (UV) part of the solar radiation spectrum. Hence, the semiconductor nanocrystal supports the absorption of visible (vis) and near-infrared (NIR) light. Up to now, various nanocrystalline materials (InP [2], CdSe [3], CdS [4,5], PbS [6], and Ge [7,8]) have been investigated as sensitizers for TiO_2_. Alternatively, the wide-gap semiconductor ZnO was also investigated, since the band gap and the energetic position of the valence band maximum and conduction band minimum of ZnO are very close to that of TiO_2_[9]. Most of these composite materials were synthesized through chemical techniques, although physical deposition, such as sputtering, is also useful. In addition, package synthesis of composite thin film is favorable for low-cost production of solar cells. Package synthesis requires a specific material design for each deposition technique, for example radio frequency (RF) sputtering [10] and hot-wall deposition [11]. The present study proposes a new composite thin film with Ge nanocrystals dispersed in Nb_2_O_5_ matrix by RF sputtering. According to the material design, based on differences in the heat of formation [10], Ge nanocrystals are thermodynamically stable in an Nb_2_O_5_ matrix, since Nb is oxidized more than Ge because the heat of formation of GeO_2_ exceeds that of Nb_2_O_5_[12]. In addition, nanocrystalline Ge dispersed in the Nb_2_O_5_ matrix may exhibit quantum-size effects due to the wide band gap of 3.4 eV in Nb_2_O_5_[13]. However, it is difficult to forecast how Nb oxides (typically NbO, NbO_2_, and Nb_2_O_5_) will be formed during the preparation process. Among these compounds, only Nb_2_O_5_ satisfies the present objective. In the current study, the composition of Ge-doped Nb-oxide (Nb-Ge-O) thin film is varied widely to produce single-phase Nb_2_O_5_ as the matrix, while retaining vis-NIR absorption due to the presence of Ge nanocrystals.

## Methods

An Nb-Ge-O thin film was prepared by RF sputtering from a composite target. Specifically, 5 × 5 mm^2^ Ge-chips were set on a 4-in.-diameter ceramic Nb_2_O_5_ target. The chamber was first evacuated to a vacuum of 1.5 × 10^−7^ Torr, and the thin film was deposited on a Corning #7059 glass substrate cooled by water. The substrate was cleaned with an acetone ultrasonic bath for 60 min to remove surface contaminations, dried using nitrogen air gun, and finally sputter-etched at an applied power of 200 W for 1 min. The distance between the target and the substrate was kept constant at 73 mm. The total gas pressure of argon or argon and additional oxygen was fixed at 2.0 × 10^−3^ Torr. RF power and deposition time were kept constant at 200 W and 90 min, and no RF bias was applied to the substrate. The Nb-Ge-O thin films thus deposited were successively post-annealed at 923 K for 60 min in a vacuum to crystallize Ge nanocrystals and the Nb-O matrix. The Nb-Ge-O thin film was structurally characterized using X-ray diffraction (XRD, Rigaku RAD-X, Rigaku Corporation, Tokyo, Japan) with Cu Kα radiation. The optical-absorption spectrum of the film was measured using UV–vis-NIR spectroscopy (Shimadzu UV5300, Shimadzu Corporation, Nakagyo-ku, Kyoto, Japan), and the composition of the film was analyzed using energy-dispersion spectroscopy (EDAX Phoenix, NJ, USA), operating at 10 kV with standard samples of KNbO_3_ to calibrate the analyzed results for elements Nb and O, and with Bi_4_Ge_3_O_12_ for element Ge. Nanoscale elemental mapping was performed using scanning transmission electron microscopy (STEM, Hitachi HD-2700, Hitachi, Ltd., Tokyo Japan) in EDX mode (EDAX model: Genesis) operating at 200 kV with an energy resolution of 150 eV. Ion milling was performed during sample preparation.

## Results and discussion

**Figure 1:**
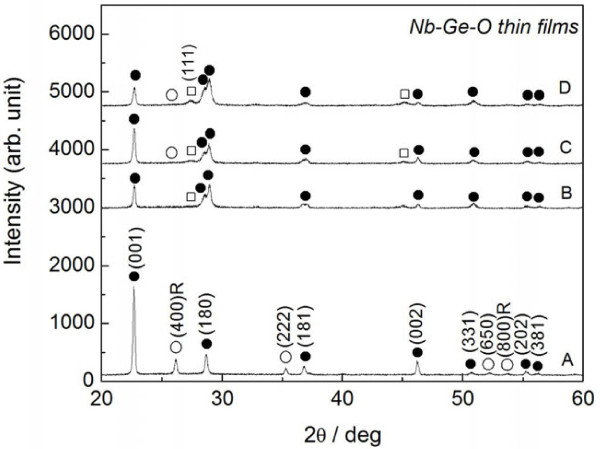
**XRD pattern of Nb-Ge-O thin films with different Ge contents.** Dots indicate orthorhombic Nb_2_O_5_, circles indicate NbO_2_, and squares indicate Ge. Labels A through D indicate Ge concentrations of 0, 1.8 at.%, 4.9 at.%, and 5.5 at.%.

**Figure 2:**
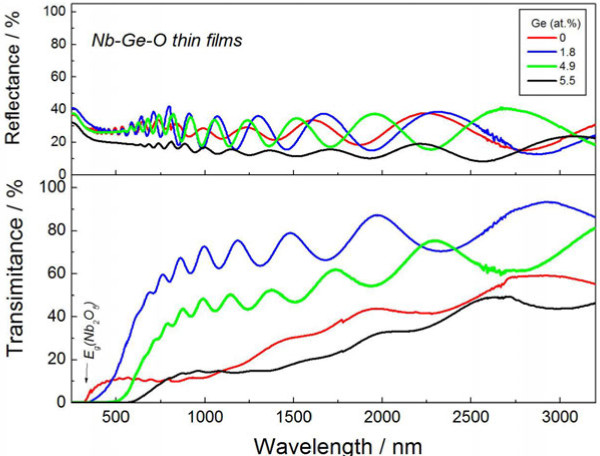
Optical transmittance and reflectance spectra of Nb-Ge-O thin films.

**Figure 3:**
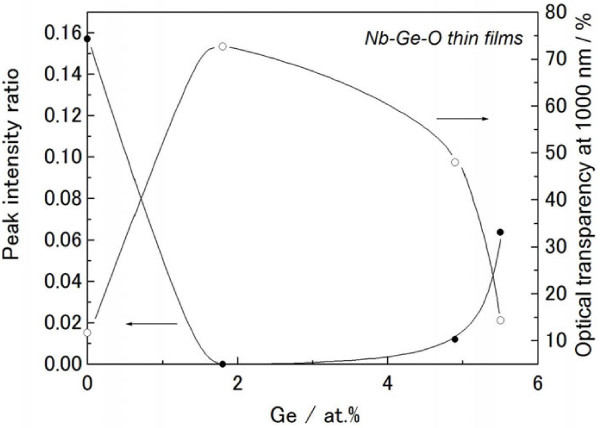
**XRD peak intensity ratio and optical transparency at 1,000 nm for Nb-Ge-O thin films.** In this case, the XRD peaks of NbO_2_ at the (400) reflection and Nb_2_O_5_ at (001) are employed.

Figure 1 presents the XRD pattern of the Nb-Ge-O thin films prepared in a pure Ar atmosphere. The as-deposited films formed an amorphous structure, and post-annealing was therefore performed to crystallize both Ge and Nb-oxide. Regarding temperature variations in a preliminary experiment (not shown here), XRD peaks of both Ge and Nb-oxide appeared at 823 K, and the peak of Ge became more prominent at 923 K. Thus, a post-annealing temperature of 923 K is employed here. At Ge = 0 at.%, a phase mixture of NbO_2_ and Nb_2_O_5_ with an orthorhombic structure is observed. The peak of NbO_2_ with a tetragonal structure disappears at a Ge concentration of 1.8 at.%, and the XRD peak of Ge appears instead. Further addition of Ge exceeding 4.9 at.% reproduces the weak NbO_2_ peak. Figure 2 presents the optical transmittance and reflectance spectra of the Nb-Ge-O thin films. The optical absorption edge of the Nb_2_O_5_ is clearly seen at 0 at.%, with relatively less transparency in the vis-IR range. This transparency is favorably improved at 1.8 at.% Ge, but further additions (4.9 at.% and 5.5 at.%) also reduce the transparency. The reflectance spectra exhibit no significant difference in vis-NIR range. Scanning electron microscopy also revealed a relatively flat appearance irrespective of the Ge concentration (not shown here). In addition, the thickness of the films increase gradually from 1,200 nm (0 at.% Ge) to 1,600 nm (5.5 at.% Ge) with increasing Ge composition. Thus, the relatively low transparency in vis-NIR range at 0 at.% and 5.5 at.% is not due to surface morphology and thicker nature. In contrast, the optical absorption edge monotonically shifts toward the long-wavelength region as the Ge concentration increases. The addition of Ge to Nb-oxide thus induces a transparency change and an absorption shift. Figure 3 plots the XRD peak intensity ratio of NbO_2_ at (400) reflection and Nb_2_O_5_ at (001) reflection, and the optical transparency at 1,000 nm for the Nb-Ge-O thin films. The XRD intensity ratio is minimized at 1.8 at.% Ge, with a maximum transparency at 1,000 nm. Hence, the transparency increases with decreasing NbO_2_ phase. In a preliminary experiment with diffused reflectance spectra, a standard powder of NbO_2_ exhibited opacity over a wide range from UV to NIR, in contrast with the visible transparency of Nb_2_O_5_. Thus, the change in the optical transparency (Figure 2) is due to the inclusion of NbO_2_.

**Figure 4:**
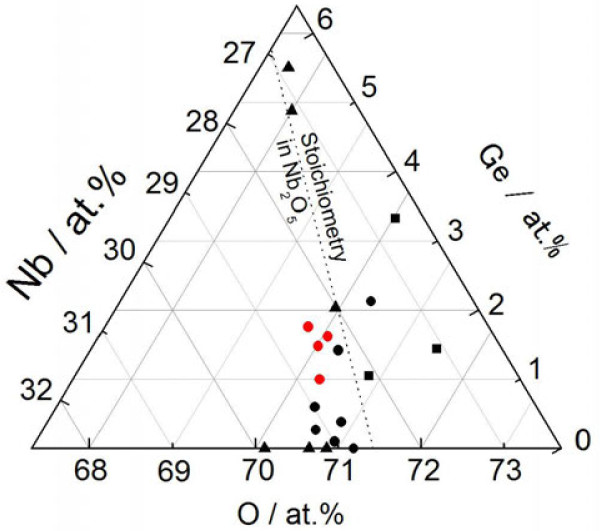
**Compositional plane of the crystal structure in Nb-Ge-O thin films.** Squares indicate phase mixture of Nb_2_O_5_ and GeO_2_, triangles indicate phase mixture of Nb_2_O_5_ and NbO_2_, black dots indicate single-phase Nb_2_O_5_, and red dots indicate phase mixture of Ge and Nb_2_O_5_.

The relation between the analyzed composition of the films and the structure derived from the XRD pattern is summarized in Figure 4. The stoichiometric composition of Nb_2_O_5_ is presented as a dotted line. The squares indicate the phase mixture of Nb_2_O_5_ and GeO_2_, which appears in a relatively high oxygen concentration range beyond the stoichiometry line of Nb_2_O_5_. In this case, the added Ge is fully oxidized, with no optical absorption shift toward the vis-NIR region. The triangles indicate the phase mixture of Nb_2_O_5_ and NbO_2_, which appears together with Ge phase in relatively high-Ge compositions and also appears in pure Nb-oxide (i.e., no Ge) in relatively lower-oxygen compositions. The black dots indicate the single phase Nb_2_O_5_, which appears in relatively low Ge compositions. The red dots indicate a phase mixture of Ge and Nb_2_O_5_, which appears in a quite narrow range of Ge concentration from 1.0 at.% to 1.8 at.%, near the stoichiometry line. The crystal structure of the Nb-Ge-O films thus changes, and single-phase Nb_2_O_5_ is selectively produced as a result of compositional optimization.

**Figure 5:**
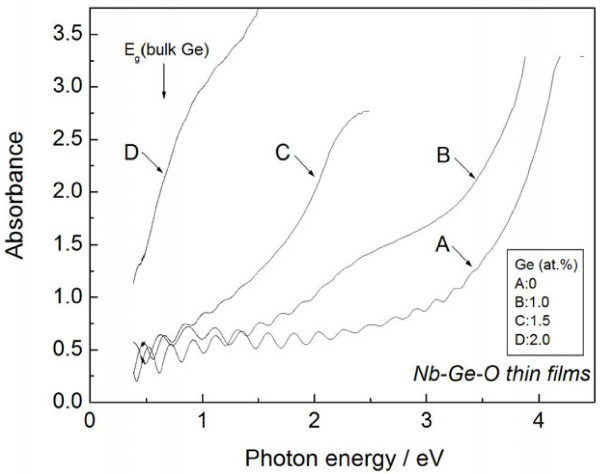
**Typical optical absorption spectra of Nb-Ge-O thin films with phase mixture of Ge and Nb**_**2**_**O**_**5**_.

Figure 5 presents typical optical absorption spectra for the Nb-Ge-O thin films with a phase mixture of Ge and Nb_2_O_5_. For comparison, the spectrum of a pure Nb_2_O_5_ thin film (A), and phase mixture of Ge, Nb_2_O_5_, and NbO_2_ (D) are also presented in the figure. An intact absorbance is employed here to exactly evaluate the absorption edge. At 0 at.% Ge, the optical absorption edge of Nb_2_O_5_ is clearly observed at 3.4 eV. The broad absorption edge shifts toward the lower-energy region as the Ge content increases. In particular, onset absorption can be confirmed at 1.0 eV with 1.5 at.% Ge, favorably covering the desirable energy region for high conversion efficiency [14]. Therefore, it should be noted that the Nb-Ge-O thin film exhibits the valuable characteristic of vis-NIR absorption.

**Figure 6:**
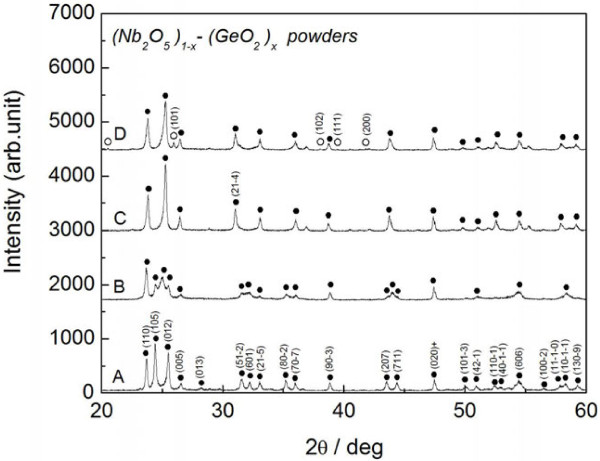
**XRD pattern of powder-synthesized (Nb**_**2**_**O**_**5**_**)**_**1− *x* **_**-(GeO**_**2**_**)**_** *x* **_**.** Dots indicate Nb_2_O_5_ and circles indicate GeO_2_. Labels A through D indicate *x* = 0, 0.08, 0.15, and 0.18.

There are two possible reasons for the shift in the optical absorption edge, forming a solid solution matrix of (Nb_2_O_5_)_1−*x*_(GeO_2_)_*x*_ and exhibiting the quantum-size effect in Ge nanocrystals. The solid solution of (Nb_2_O_5_)_1−*x*_(GeO_2_)_*x*_ was investigated first. Nb-Ge-O thin film often contains multiple phases, and it is then difficult to focus on the matrix characteristics. The (Nb_2_O_5_)_1−*x*_(GeO_2_)_*x*_ solid solution is therefore powder-synthesized here. This system was found to form a solid solution [15,16], but the energy band gap of this system is still unclear. The fundamental properties of the solubility range of Ge and the energy band gap were therefore investigated to clarify whether the ternary solid solution exhibits vis-NIR absorption. Figure 6 presents the powder XRD pattern of the (Nb_2_O_5_)_1−*x*_(GeO_2_)_*x*_ system. In the powder synthesis, standard powders of orthorhombic Nb_2_O_5_ (3 N pure) and GeO_2_ (4 N pure) were used as a starting materials. The powders were weighed for the desired composition, mixed in an agate mortar, and pressed at 49 MPa to promote a solid-state reaction. Heat treatment was performed at 1,273 K for 96 h in air to achieve thermal equilibrium, followed by water-quenching to maintain the solubility range at the synthesis temperature. The sample, thus heat-treated, was well hardened and then crushed into powder for the following experiment setup. The composition of the (Nb_2_O_5_)_1−*x*_(GeO_2_)_*x*_ system, thus powder-synthesized, is the nominal value. In the preliminary experiment, a mass reduction during the heat treatment was found to be less than 1% in standard powders of Nb_2_O_5_ and GeO_2_, suggesting a quite small amount of sublimation. The nominal content of Ge is therefore employed here as a composition of the production. At *x* = 0, all of the XRD peaks are assigned to the monoclinic Nb_2_O_5_. The XRD peak of GeO_2_ appears at *x* = 0.18, forming a phase mixture of GeO_2_ and Nb_2_O_5_. It is suggested that the present sample possibly forms a solid solution of (Nb_2_O_5_)_1-*x*_(GeO_2_)_*x*_ at compositions below *x* = 0.18.

**Figure 7:**
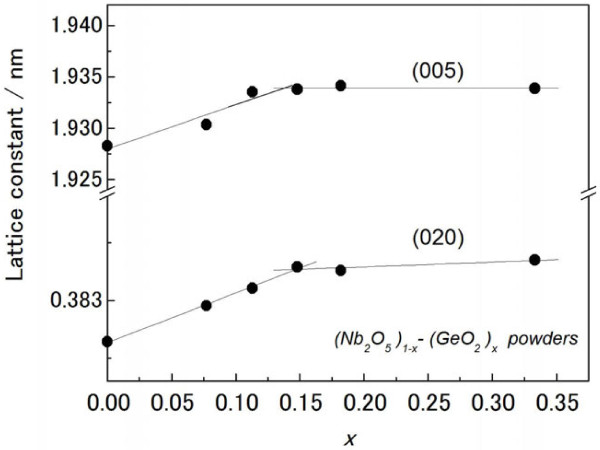
**Lattice constant of powder-synthesized (Nb**_**2**_**O**_**5**_**)**_**1− *x* **_**-(GeO**_**2**_**)**_** *x.* **_

Next, the solubility limit of Ge in Nb_2_O_5_ is determined through variations in the lattice constant. Figure 7 plots the lattice constant of the (Nb_2_O_5_)_1−*x*_(GeO_2_)_*x*_ solid solution as a function of *x*. Here, the lattice constant of the orthorhombic system is estimated from the (005) and (020) reflections. It is clearly seen in the figure that the lattice constant first increases linearly in proportion to *x* and then becomes nearly constant irrespective of *x* in the range exceeding 0.14 in both the (005) and (020) reflections. According to Vegard's law [17], an on-setting composition *x* deviating from linearity is regarded as the solubility limit of GeO_2_ in Nb_2_O_5_. This was therefore determined to be 0.14 at 1,273 K. This result strongly suggests that the Nb_2_O_5_ phase in the Nb-Ge-O thin film (Figure 4) may have formed a solid solution of (Nb_2_O_5_)_1−*x*_(GeO_2_)_*x*_. Subsequently, optical absorption of the (Nb_2_O_5_)_1−*x*_(GeO_2_)_*x*_ thus powder-synthesized should be investigated regardless of whether the solid solution exhibits vis-NIR absorption.

**Figure 8:**
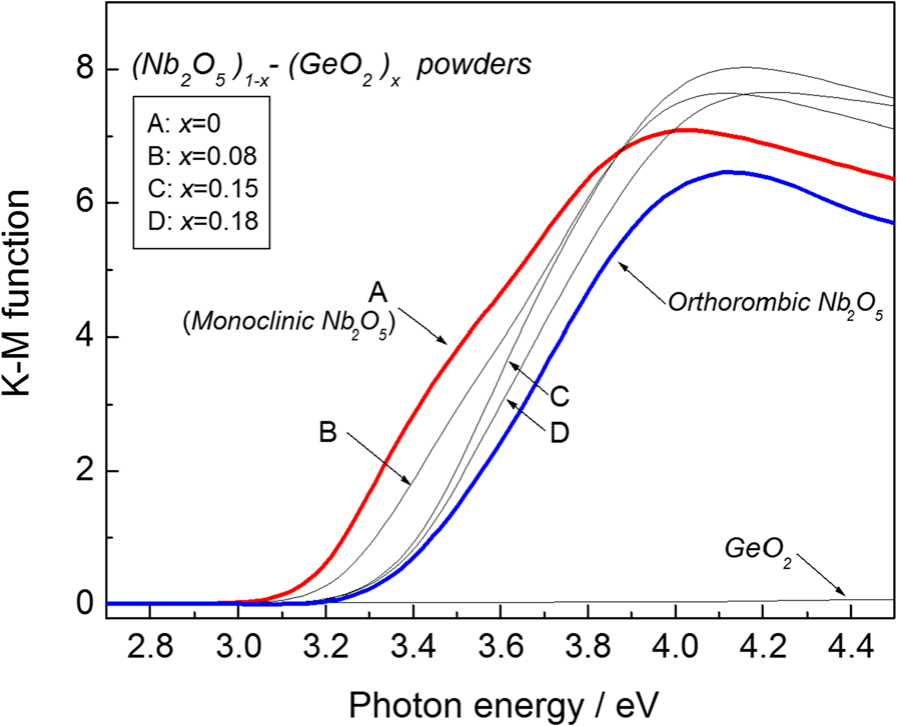
**Typical optical absorption spectra of powder-synthesized (Nb**_**2**_**O**_**5**_**)**_**1- *x* **_**-(GeO**_**2**_**)**_** *x.* **_

Figure 8 plots the optical absorption spectra of the powder-synthesized (Nb_2_O_5_)_1−*x*_(GeO_2_)_*x*_ solid solution. These spectra are derived from the Kubelka-Munk function [18]. For comparison, the spectrum of orthorhombic Nb_2_O_5_ and GeO_2_ is also shown. It is clearly seen that the GeO_2_ is fully transparent over the measured range from 2.7 to 4.5 eV, whereas the optical absorption edge of the monoclinic (Nb_2_O_5_)_1−*x*_(GeO_2_)_*x*_ can be clearly observed at 3.1 eV at *x* = 0 and shifts toward the higher-energy region as *x* increases. In addition, the band gap of orthorhombic Nb_2_O_5_ is higher than that of monoclinic Nb_2_O_5_. From these results, orthorhombic Nb_2_O_5_-phase in the Nb-Ge-O thin films may have formed a solid solution of (Nb_2_O_5_)_1−*x*_(GeO_2_)_*x*_ during post-annealing at 923 K but did not exhibit vis-NIR absorption. The quantum-size effect in the Ge nanocrystals was also investigated as another possible reason for the absorption shift (Figure 5).

**Figure 9:**
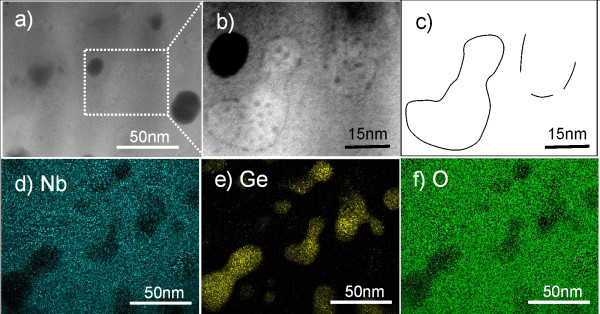
**Direct observation of Nb-Ge-O thin film with phase mixture of Ge and Nb**_**2**_**O**_**5**_**, containing 1.5 at.% Ge.** (**a**) HAADFSTEM image; (**b**) enlarged image for easier viewing; (**c**) schematic image; (**d**) elemental mapping of Nb (blue), (**e**) Ge (yellow), and (**f**) O (green).

Figure 9a presents an image resulting from high-angle annular dark-field scanning transmission microscopy (HAADF-STEM) of the Nb-Ge-O thin film with a phase mixture of Ge and Nb_2_O_5_, containing 1.5 at.% Ge. In this case, Nb-oxide is compositionally optimized to form a single phase Nb_2_O_5_. The difference in atomic number can be determined by the observed contrast in the HAADF-STEM image. Several black grains are seen in the image in Figure 9a, but this black contrast is not due to the difference in atomic number, since there is no compositional change near the black grains on the STEM-EDX elemental mapping of the sample, based on X-ray detection of Nb L (blue) (Figure 9d) and O K (green) (Figure 9f). Hence, these black grains probably indicate a structural defect in the film. In another area with a gray color (Figure 9a), the graphic contrast is too weak to determine the difference in atomic number. The image contrast is thus emphasized in the enlarged Figure 9b for easier viewing. Gourd-shaped grains, which are schematically illustrated in Figure 9c, can be seen in the image. The gourd-shaped grains mainly indicate the absence of elemental Nb and O (Figures 9d,f) and the presence of Ge [Ge K (yellow), Figure 9e. It is thus determined that the gourd-shaped grains (Figure 9b) are dominantly nanocrystalline Ge. The other region is widely covered with the elements Nb and O (Figures 9d,f), reasonably assumed to form Nb-oxide. It is therefore determined that isolated Ge nanocrystals are dispersed in the Nb_2_O_5_ matrix. The mean grain size of the Ge nanocrystals is estimated to be 11 nm, according to the XRD result using Scherrer's equation [19]. This is too small to exhibit the quantum-size effect because of the exciton Bohr radius of 24.3 nm in Ge [20]. Assuming an infinite potential barrier, optical gap of the Ge nanocrystals with the mean size of 11 nm is calculated to be 1.1 eV using Brus model [21]. This value is close to the onset absorption of 1.0 eV at 1.5 at.% Ge (Figure 5). Ge nanocrystals embedded in silica matrix also exhibits similar optical absorption spectrum, with the mean size of 6.0 nm estimated from direct observation using high-resolution transmission microscopy [22]. Consequently, the shift of the optical absorption edge (Figure 5) is reasonably due to the presence of Ge nanocrystals embedded in the Nb_2_O_5_ matrix.

These results indicate that the Nb-Ge-O thin films can selectively produce the Nb_2_O_5_ matrix and the vis-NIR absorption simultaneously, despite the package synthesis by RF sputtering. One-step synthesis of a composite package with Ge nanocrystals dispersed in Nb_2_O_5_ matrix therefore has the potential to yield low-cost production of next-generation solar cells.

## Conclusions

A new composite thin film with Ge nanocrystals dispersed in Nb_2_O_5_ matrix has been proposed as a candidate material for quantum-dot solar cells. It should be pointed out that single-phase Nb_2_O_5_ appears in a restricted composition range from 1.0 to 1.8 at.% Ge as a result of compositional optimization based on the Ge chip number and oxygen ratio in argon. Furthermore, the optical absorption edge shifts toward the lower-photon-energy region as the Ge content increases. In particular, onset absorption can be confirmed at 1.0 eV with 1.5 at.% Ge, favorably covering the desirable energy region for high conversion efficiency. Elemental mapping indicates that the isolated Ge nanocrystals are dispersed in the Nb_2_O_5_ matrix. Thus, two valuable characteristics, the selective production of Nb_2_O_5_ and vis-NIR absorption, are simultaneously retained in Nb-Ge-O thin films.

## Competing interests

The author declares that there are no competing interests.

## Authors' information

SA is a group leader of Research Institute for Electromagnetic Materials.
